# Historical changes in the contents and compositions of fibre components and polar metabolites in white wheat flour

**DOI:** 10.1038/s41598-020-62777-3

**Published:** 2020-04-03

**Authors:** Alison Lovegrove, Till K. Pellny, Kirsty L. Hassall, Amy Plummer, Abigail Wood, Alice Bellisai, Alexandra Przewieslik-Allen, Amanda J. Burridge, Jane L. Ward, Peter R. Shewry

**Affiliations:** 1 Plant Science Department, Rothamsted Research, Harpenden, Hertfordshire AL5 2JQ UK; 2 Computational and Analytical Sciences Department, Rothamsted Research, Harpenden, Hertfordshire AL5 2JQ UK; 3 Life Sciences, University of Bristol, 24 Tyndall Avenue, Bristol, BS8 1TQ UK

**Keywords:** Analytical biochemistry, Plant breeding

## Abstract

Thirty-nine UK adapted wheat cultivars dating from between 1790 and 2012 were grown in replicated randomised field trials for three years, milled, and white flour analysed for the contents of dietary fibre components (arabinoxylan and β-glucan) and polar metabolites (sugars, amino acids, organic acids, choline and betaine) to determine whether the composition had changed due to the effects of intensive breeding. The concentrations of components varied between study years, indicating strong effects of environment. Nevertheless, some trends were observed, with the concentrations of arabinoxylan fibre and soluble sugars (notably sucrose, maltose and fructose) increasing and most amino acids (including asparagine which is the precursor of acrylamide formed during processing) decreasing between the older and newer types. The concentration of betaine, which is beneficial for cardio-vascular health, also increased. The study therefore provided no evidence for adverse effects of intensive breeding on the contents of beneficial components in wheat flour.

## Introduction

Scientific plant breeding has been immensely successful in increasing the yield and improving the performance of wheat. For example, Mackay *et al*.^1^ calculated that about 88% of the gain in yield of winter wheat in the UK between 1981 and 2007, from about 6 to 8 tonnes, is attributable to genetic improvement. However, modern cultivars have lower genetic diversity than older cultivars and land races, particularly in the A and B subgenomes^2^. It has also been suggested that modern plant breeding, with emphasis on high yield (which effectively reflects starch content) and, in the case of wheat, on gluten protein content, may have impacts on grain composition which result in negative effects on health^3,4^. In fact, Kasarda^5^ reported a decline in the protein content of wheats grown in the Northern Plains of the USA, and we have reported similar decreases in the protein contents of UK wheats^6^. Because starch constitutes about 80% of the grain, a decrease in protein content with increasing yield would be expected and is often ascribed to “yield dilution”. Yield dilution may also contribute to the decreases in the mineral micronutrients (iron and zinc) that have occurred since the introduction of dwarfing genes in the 1960s^7,8^. However, other effects of dwarfing genes, for example on mineral nutrient uptake and partitioning, may also have contributed^6^.

Effects of intensive breeding on other bioactive components are less clear. Shewry *et al*.^9^ compared the contents of phytochemicals in a global collection of 146 bread wheat genotypes in relation to their dates of registration. No clear relationships were identified, but only a single set of samples were analysed and these were grown on the same site in Hungary, which was outside the area of adaptation of many of the lines. However, comparisons of smaller numbers of “old and recent” adapted cultivars showed no difference in the total contents of phenolics in durum or bread wheats, although the composition was more diverse in the older cultivars^10,11^. A study of eight modern and 7 older Italian durum wheats cultivars showed no differences in contents of arabinoxylan and β-glucan in wholemeal and semolina, but higher arabinoxylan solubility in modern cultivars^12^. These studies have largely focused on wholemeal samples, which are richer in bioactive components. However, the most widely consumed foods in many countries, including the UK, are produced from white flour and hence the relevance to human health of analyses carried out on whole grains is debatable. We have therefore determined historical trends in the composition of white flour of bread wheat, by comparing 39 varieties which are adapted to the UK where they have been grown commercially over the past 200 years.

## Results

### Selection and genetic diversity of wheat cultivars

**Figure 1:**
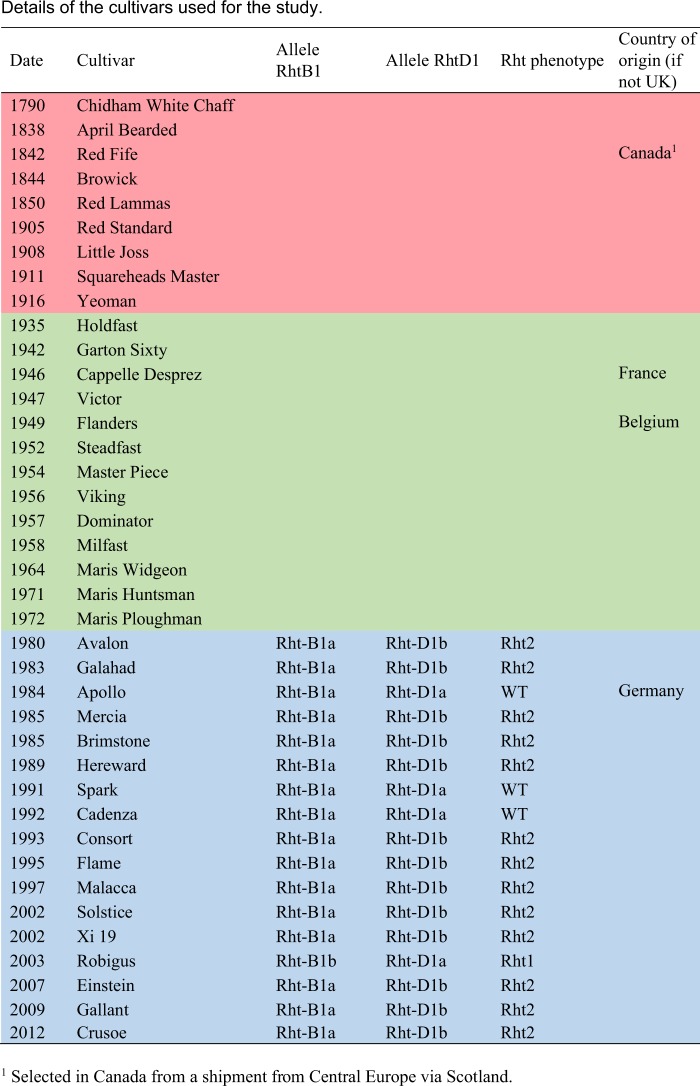
Details of the cultivars used for the study. Three groups are coloured as in the figures. Red, empirical selection (1790–1916); green, early breeding (1935–1972), blue, modern breeding (1980–2012). ^1^Selected in Canada from a shipment from Central Europe via Scotland.

A series of 39 wheat cultivars (including cultivars and earlier land races, all referred to as cultivars here) was selected to represent the diversity in wheat grown in the UK since 1790 (Fig. 1). All were either winter type, or winter-hardy, and hence routinely grown as winter wheats. They include 9 out of the 11 cultivars grown in the Broadbalk continuous winter wheat experiment (which has been grown at Rothamsted since 1843^13^). All selected cultivars had been grown commercially in the UK, with some being regarded as “landmark varieties”, and all except 4 were bred in the UK (Fig. 1). Hence, they can be regarded as well adapted to the UK climate. The cultivars are divided into three groups, which are colour coded and represent stages in the development of wheat breeding. Group 1 comprises 9 cultivars which were released between 1790 and 1916. During this period selections were made from landraces and from populations from early crosses, but selection was empirical without an understanding of genetic mechanisms. The second group comprises 13 cultivars released between 1935 and 1972, which represent the increasing application of scientific theory to wheat breeding. The third group comprises 17 cultivars released between 1980 and 2012 which represent the products of modern breeding technologies (with investment in wheat breeding being stimulated in the UK by an increased demand for homegrown wheat following the accession into the European Union in 1973). The major scientific advance during this period was the introduction in the 1970s of the “green revolution” dwarfing genes which increase the harvest index and hence yield. Consequently, the *Rht2* dwarfing gene is present in 13 of these cultivars and the *Rht1* dwarfing gene in one (Fig. 1). In addition, increasing use was made of “alien introgression”, for example, Xi19, Cadenza, Robigus and Crusoe all contain introgressions from *Triticum dicoccoides*, and of new technologies to increase the efficiency of breeding (such as doubled haploid production). These three groups are therefore termed “empirical selection”, “early breeding” and “modern breeding”. The pedigrees of the cultivars, where known, are shown in Supplementary Fig. S1.

**Figure 2:**
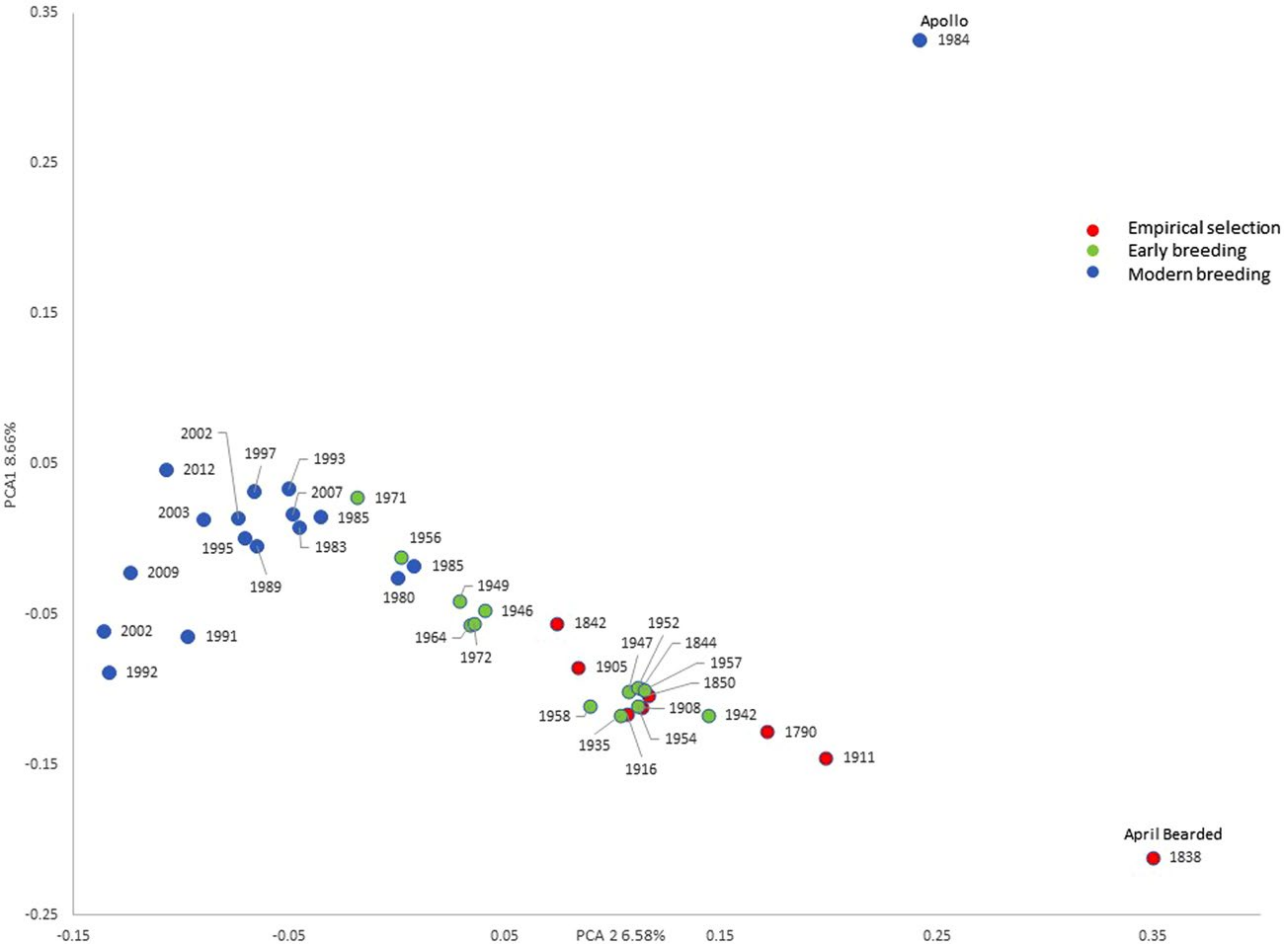
Genomic relationships of the 39 cultivars, illustrated by Principal Component Analysis of markers determined using the Axiom HD Genotyping Array (comprising 819,571 SNP markers). Cultivars are coloured to indicate three groups representing stages in wheat breeding: empirical selection (1790–1916), early breeding (1935–1972) and modern breeding (see Fig. 1). Two outliers indicated, labelled (April Bearded, 1884; Apollo, 1986) are discussed in the text.

The broad genetic relationships between the cultivars were initially determined using the Axiom Wheat HD Genotyping Array, comprising 819,571 SNP markers, and the data analysed by principal component analysis (PCA) (Fig. 2). Comparison of PCs 1 and 2, which accounted for 8.66% and 6.58% of the total variation, respectively, showed that the cultivars released since 1980 were more closely related to each other than those released before 1980. Two cultivars are clearly separated from the others: April Bearded (1838) and Apollo (1984) (labelled in Fig. 2). In both cases the separations may result from introgressions. Wider studies have shown that April Bearded has DNA in common with *Triticum aestivum* ssp. *compactum* (also called club wheat) while Apollo has DNA in common with rye (presumably derived from Triticale which is present in the pedigree, see Supplementary Fig. S1) (authors’ unpublished results). This analysis, and plots of further PCs (PC3 5.85%, PC4 5.1%, PC5 3.88%, PC6 3.68%) (not shown), indicate that the recent cultivars are less genetically diverse that the older cultivars.

### Crop and grain phenotyping

Decreases in the height of wheat cultivars grown in the UK over the past century are well-documented^14^ and have been reported previously for the cultivars in the present study^6^. The use of small experimental plots, and the difficulty in harvesting some of the older cultivars, precluded the determination of grain yield, or of grain number which is one of the two determinants of yield. However, grain weight and kernel diameter both showed significant differences between cultivar groups (F_2,225_ = 106.41, p < 0.001 and F_2,225_ = 115.18, p < 0.001). Furthermore, although these group differences differed between experimental years (significant interaction effects, F_4,225_ = 35.93, p < 0.001 and F_4,225_ = 16.32, p < 0.001), both grain weight and kernel diameter tended to be higher in the more recent (post-1935) cultivars (Table 1, Supplementary Table S1, Supplementary Fig. S2). Similar studies have shown that the grain weight of wheat cultivars did not increase in the USA over the period 1919–1987^15^ or in Canada between 1947 and 1992^16^. Grain hardness also showed significant differences between groups (F_2,225_ = 504.36, p < 0.001) with more recent groups, on average, having a higher grain hardness (Table 1, Supplementary Table S1). This is probably due to increased emphasis on breeding for breadmaking quality.

**Table 1:** ANOVA of the treatment effects for Single Kernel Characterisation System (SKCS) measurements; grain kernel weight, diameter and hardness index.

Variable	Cultivar/Time				(Group\Cultivar)/Time							
	Cultivar	Time.Cultivar	Cultivar	Time.Cultivar	Group	Time.Group	Group.Cultivar	Time.Group.Cultivar	Group	Time.Group	Group.Cultivar	Time.Group.Cultivar
		F statistic	F statistic	p-value	p-value	F statistic	F statistic	F statistic	p-value	p-value	p-value	p-value
Kernel weight	52.29	52.07	1.76E-91	0.00E + 00	106.41	35.93	49.28	52.96	2.99E-33	3.31E-23	1.92E-87	0.00E + 00
Kernel diameter	31.23	3.83	4.67E-70	4.66E-15	115.18	16.32	26.57	3.14	3.59E-35	9.39E-12	2.05E-62	5.31E-11
Hardness index	89.36	21.33	3.16E-115	0.00E + 00	504.36	52.26	66.31	19.62	7.22E-84	4.35E-31	2.70E-100	0.00E + 00

All treatment effects were tested on 225 residual degrees of freedom. Table shows both the F statistic (representing the size of the effect) and the p-value (the statistical significance of the effect). See also Supplementary Fig. S2; box whisker plot of data with group means indicated by black asterisk (∗).

### Flour composition

In order to determine whether there were differences in the composition of white flour, which accounts for about 90% of the flour used for breadmaking in the UK (http://www.nabim.org.uk/statistics/), white flour fractions were prepared and analysed for two groups of components: dietary fibre (arabinoxylan and β-glucan) and polar metabolites (comprising mainly amino acids, sugars and small oligosaccharides, arabinogalactan peptide (AGP), choline and betaine). These components were selected because wheat is the major source of dietary fibre in the UK diet^17^ and the metabolites include components which contribute to health. The full datasets for individual components are given in Supplementary Table S2.

Statistical analysis of the data using ANOVA shows highly significant differences between individual cultivars for all measured variables (Supplementary Table S3). Furthermore, strong differences are observed between the three groups which, with 2 exceptions (choline and arabinose equivalents in AGP), are greater than the differences observed between the individual cultivars within each group. Although it is clear that environmental factors affect the content and composition of fibre and metabolites, this interaction is small compared with the main effect of genotype.

**Figure 3:**
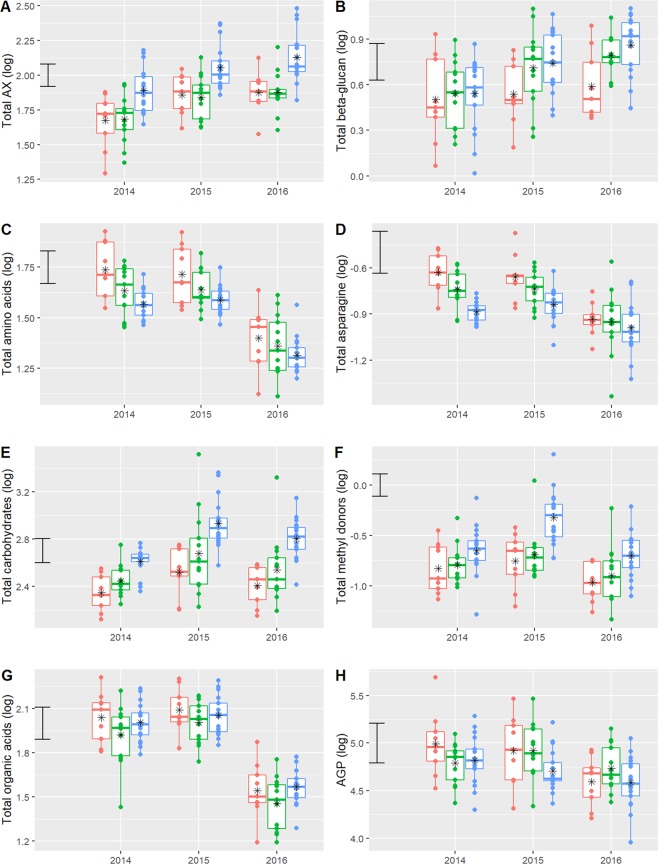
Box and whisker plots of the contents of fibre components and polar metabolites in white flour of the 39 cultivars. Cultivars are grouped to represent stages in wheat breeding: red, empirical selection (1790–1916); green, early breeding (1935–1972), blue, modern breeding (1980–2012) (see Fig. 1). Each point represents the mean of 3 biological replicates. Error bar gives the least significance difference (LSD) between genotype means. (**A**) arabinoxylan (AX); (**B**), β-glucan; (**C**), total free amino acids; (**D)**, free asparagine; (**E**), total carbohydrates (mono-, di- and tri-saccharides); (**F**), total methyl donors (betaine and choline); (**G**), total organic acids; **(H**), arabinogalactan peptide (AGP).

In order to focus on differences between the cultivars, mean values for eight individual components or groups of components over the three years are summarised in Table 2; Fig. 3 and Supplementary Table S3.

**Table 2:** ANOVA of the treatment effects for fibre components and polar metabolite variables.

**Row number**	**Variable**	**Transformation**	Fig. 3	**Cultivar\Time**				**Group\Cultivar\Time**							
				***Cultivar***	***Time.Cultivar***	***Cultivar***	***Time.Cultivar***	***Group***	***Time.Group***	***Group.Cultivar***	***Time.Group.Cultivar***	***Group***	***Time.Group***	***Group.Cultivar***	***Time.Group.Cultivar***
				*F statistic*	*F statistic*	*p-value*	*p-value*	*F statistic*	*F statistic*	*F statistic*	*F statistic*	*p-value*	*p-value*	*p-value*	*p-value*
1	Arabinoxylan	Log	A	29.57	2.27	3.63E-67	1.80E-06	212.17	1.06	19.43	2.34	3.75E-52	3.76E-01	1.92E-50	1.15E-06
2	Total beta glucan	Log	B	17.40	1.31	8.84E-48	6.51E-02	36.47	5.71	16.34	1.07	2.05E-14	2.16E-04	1.36E-44	3.49E-01
3	Total amino acid	Log	C	10.82	1.14	7.07E-33	2.25E-01	44.03	1.65	8.98	1.12	8.03E-17	1.63E-01	5.22E-27	2.70E-01
4	Asparagine	Log	D	4.37	1.40	1.67E-12	3.25E-02	27.02	3.34	3.11	1.29	3.18E-11	1.11E-02	1.60E-07	8.48E-02
5	Total carbohydrates	Log	E	31.54	2.80	1.10E-69	2.24E-09	241.95	4.30	19.85	2.72	2.18E-56	2.29E-03	3.40E-51	1.08E-08
6	Total methyl donors	Log	F	30.76	2.25	1.06E-68	2.41E-06	152.41	11.54	24.00	1.73	2.53E-42	1.58E-08	5.33E-58	1.29E-03
7	Total organic acids		G	8.97	1.55	9.27E-28	7.27E-03	16.34	0.97	8.56	1.59	2.40E-07	4.26E-01	8.08E-26	5.94E-03
8	AGP		H	7.18	1.41	2.79E-22	2.88E-02	9.53	5.04	7.05	1.21	1.07E-04	6.59E-04	3.13E-21	1.52E-01

Fibre components; arabinoxylan (AX) and total β-glucan; and polar metabolites; total free amino acids, free asparagine, total carbohydrates (mono-, di- and tri-saccharides), total methyl donors (betaine and choline), total organic acids and arabinogalactan peptide (AGP).

All treatment effects were tested on 221 residual degrees of freedom. Table shows both the F statistic (representing the size of effect) and the p-value (the statistical significance of the effect). See also Fig. 3; box whisker plot of data with group means indicated by black asterisk (∗).

Table 2 (rows 1 and 2) and Fig. 3 (parts A and B) show the amounts of AX and β-glucan, the two major dietary fibre components in white flour. Both components show significant differences between cultivar groups (‘Group’ column), (F_2,221_ = 212.17, p < 0.001 and F_2,221_ = 36.47, p < 0.001). Moreover, the difference between groups is consistent over study years for AX, (row 1, ‘Time.Group’ column) (F_4,221_ = 1.06, p = 0.3764), despite changes in the differences within each group over the study years (‘Time.Group.Cultivar’ column) (F_72,221_ = 2.34, p < 0.001). By contrast, the group differences for β-glucan are not consistent over study years (row 2, ‘Time.Group’ column) (F_4,221_ = 5.71, p < 0.001). The associated cultivar means (shown in Table 2, Fig. 3 parts A and B and Supplementary Table S4) highlight the stronger trend in the amount of AX (clearly higher in recent cultivars) compared to the amount of β-glucan, which varies more and shows weaker trends except for being low in the early cultivars.

**Figure 4:**
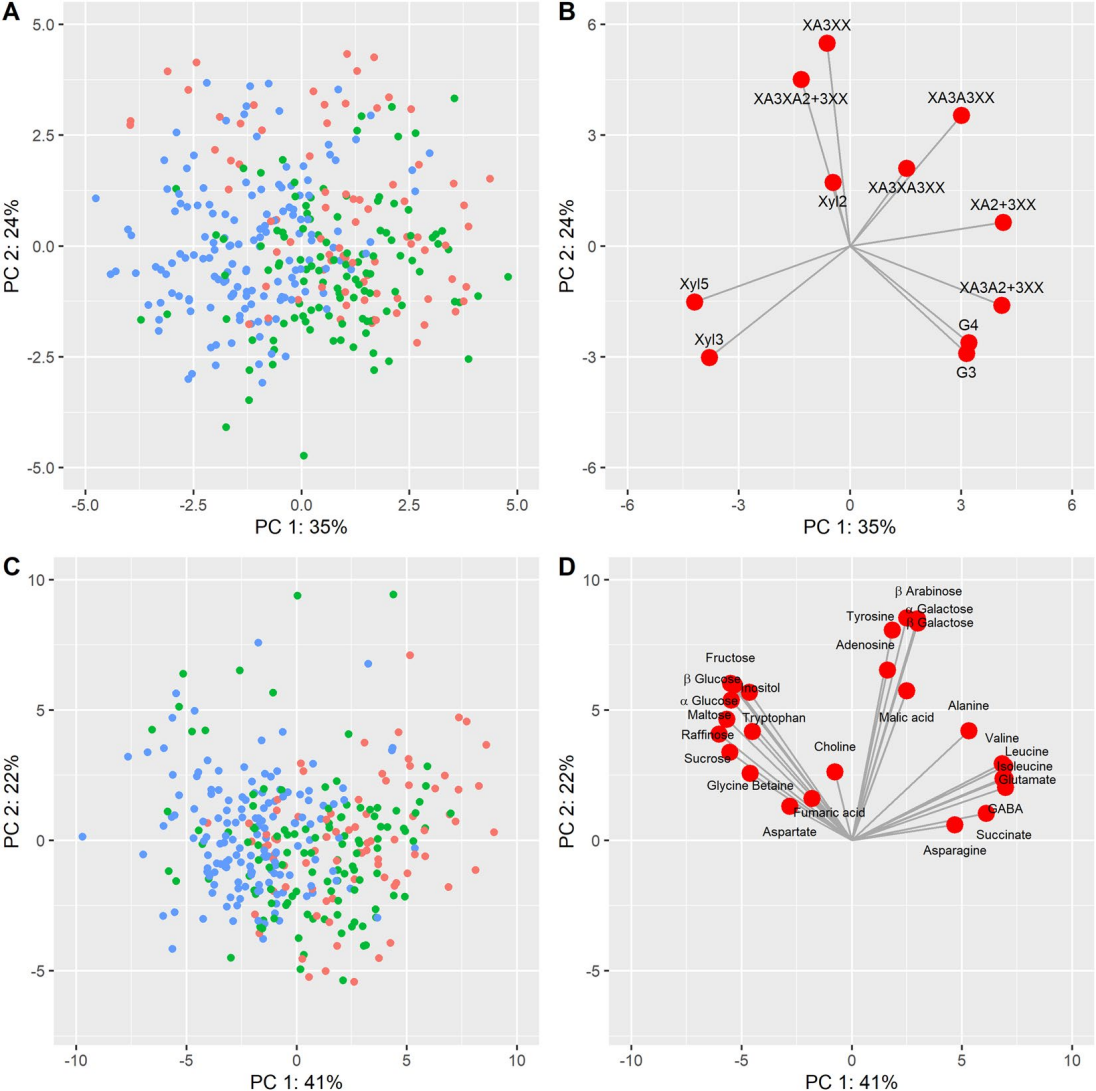
Principle Component Analysis (PCA) of fibre composition (**A,B**) and polar metabolite composition **(C,D**) in white flour of the 39 cultivars. Each point represents the mean of 3 biological replicates. Data points in (**A,C**) are coloured to indicate three groups of cultivars based representing stages in wheat breeding: red, empirical selection (1790–1916); blue, early breeding (1935–1972); green, modern breeding (see Fig. 1). (**A**) scores plot of proportions of oligosaccharides released by digestion of arabinoxylan (AXOS) and β-glucan (G3 and G4 GOS) with endoxylanase and lichenase, respectively; (**B**), loadings plot showing the contributions of AXOS and GOS to the separation in (**A,C**), scores plot of abundances of polar metabolites; D loadings plot showing the contributions of individual metabolites to the separation shown in (**C**).

Changes in the structure and composition of the dietary fibre fraction were also studied by Principal Component Analysis (PCA), comparing the proportions of arabinoxylan oligosaccharides (AXOS) released by digestion of AX with endoxylanase and of gluco-oligosaccharides (G3 and G4 GOS) released by digestion of β-glucan with lichenase (β-glucanase). Figure 4 (parts A and B) compares PCs 1 and 2, which together account for 59% of the total variation. Although the three groups of cultivars clearly overlap, partial separation is observed with the modern cultivars clustered in the left-hand part of the graph. The loadings plot (Fig. 4B) shows that the separation along PC1 is associated with Xyl5 and Xyl3 (positively associated with modern breeding) and XA2 + 3XX, XA3A2 + 3XX, XA3A3XX, G3 and G4 (positively associated with empirical selection and early breeding).

Table 2 (rows 3 and 4) and Fig. 3 (parts C and D) show the concentrations of total free amino acids and free asparagine, the latter being of interest to grain processors as it is a precursor, and usually the limiting factor, for acrylamide formation during wheat processing^18^. The concentrations of both show downward trends which are associated with strong statistical differences between groups ((‘Group’ column, F_2,221_ = 44.03, p < 0.001 andF_2,221_ = 27.02, p < 0.001)). These are consistent over study years for total free amino acids (‘Time.Group’ column, F_4,221_ = 1.65, p = 0.1630) but less so for free asparagine (‘Time.Group’ column, F_4,221_ = 3.34, p = 0.0111). Similar trends are observed for the concentrations of most of the other individual amino acids (Supplementary Tables S3 and S4).

By contrast, the concentrations of total monosaccharides and small oligosaccharides (called total carbohydrates in Fig. 3) are generally higher in recent cultivars (Table 2 row 5 and Fig. 3 part E) with strong statistical differences being observed between groups (although these are not consistent over study years) ((‘Group’ column, F_2,221_ = 241.95, p < 0.001), (‘Time.Group’ column, F_4,221_ = 4.30, p = 0.0023)). This fraction comprises sucrose, raffinose, maltose, glucose, fructose, galactose and arabinose, with sucrose, maltose and fructose showing the clearest increases with time (Supplementary Tables S3 and S4).

Table 1 (row 6) and Fig. 3 (part F) show total methyl donors, which comprises choline and betaine (glycine betaine), and shows highly significant differences between cultivar groups (‘Group’ column), (F_2,221_ = 152.41, p < 0.001). Betaine is generally present at 10-fold higher concentrations in wheat than choline^19^, and the increase in total methyl donors shown in Table 2 and Fig. 3 is due to higher contents of betaine in more recent cultivars, with little change in the contents of choline (Supplementary Tables S3 and S4).

Finally, Table 1 (rows 7 and 8) and Fig. 3 (parts G and H) show total organic acids and the wheat arabinogalactan peptide (AGP). The organic acids comprise fumaric acid, succinic acid and malic acid. The concentrations of these components show strong differences between the groups of cultivars (‘Group’ column, F_2,22,1_ = 16.34, p < 0.001), albeit much weaker than for the previously discussed components. AGP is a short (15 amino acid) peptide which is *o*-glycosylated, probably on three hydroxyproline residues^20^. It accounts for about 0.4% of white flour^21^ and is readily fermented by faecal bacteria *in vitro*^22^, indicating that it may have prebiotic properties *in vivo*. The concentration shown in Fig. 3 (part H) is the mean of galactose and arabinose equivalents determined by NMR spectroscopy (Supplementary Table S3). Although significant differences between cultivar groups are observed (‘Group’ column, F_2,221_ = 9.53, p < 0.001), no clear trends across the groups of cultivars are evident.

To confirm the overall trends discussed above, the full datasets for all metabolites over the three years were compared by PCA analysis. Figure 4 (parts C and D) compares PCs 1 and 2, which together account for 63% of the total variation. Although there is overlap between the groups of cultivars based on release date, some separation between the older and most recent groups is observed (Fig. 4, part C). The loadings plot (Fig. 4, part D) shows that this separation is related to lower and higher concentrations of amino acids and sugars, respectively, in the most recent cultivars. This separation is confirmed by the individual ANOVAs (Supplementary Table S3), where highly significant group effects are seen, particularly in sucrose, tryptophan, betaine, fumaric acid and raffinose.

## Discussion

The cultivars compared here were selected because they have been widely grown in the UK. Hence, the differences observed should not be related to their degree of adaptation. Nevertheless, all components measured were highly affected by the environment, as shown by the comparison of samples from three harvests shown in Supplementary Table S4. Therefore, in order to identify broad trends, it was decided to calculate the means of the contents determined for the three years for individual cultivars, and then the means of three groups of cultivars selected to represent different stages of wheat breeding. When this was done, clear trends were observed for some components, as summarised in Fig. 3, and Table 2

The components measured included several which are considered to contribute to effects on the health of consumers. The most important of these for most consumers is dietary fibre, as bread provides about 20% of the total daily intake in the UK, and white bread about half of this^17^. Hence, the higher contents of arabinoxylan, the major dietary fibre component, in modern cultivars are particularly noteworthy. By contrast, fermentable sugars may have beneficial or adverse effects. Raffinose and fructose have been defined as FODMAPs (fermentable oligo-, di- and monosaccharides and polyols), a group of compounds which have been implicated in causing discomfort in patients with irritable bowel syndrome (IBS)^23^. By contrast, these sugars and AGP may also have beneficial prebiotic effects in healthy individuals. The biochemical basis for the increased concentrations of sugars is not known, but it could relate to the higher levels of starch synthesis and accumulation. Similarly, the lower concentrations of amino acids in the recent cultivars could relate to their lower content of protein, which decreased from about 16.9% to 12.5% in the sample sets from years 1 and 2 (determined as N × 6.25 and reported by Shewry *et al*.^6^).

Finally, betaine and choline are biosynthetically related components which are considered to be beneficial for cardio-vascular health, by acting as methyl donors in the homocysteine cycle^24^. Wheat is a particularly rich source of these compounds, which together account for about 1.5 to 3 mg/g dry wt in wholemeal^19^. The increased concentration of betaine in the samples could therefore contribute to greater health benefits.

The conclusion from this study is, therefore, that there is no evidence that the health benefits of white flour from wheat grown in the UK have declined significantly over the past 200 years. In fact, increasing trends in several components, notably the major form of dietary fibre (arabinoxylan) are observed. This is despite great increases in the yields of wheat grown over this period. However, there are strong environmental effects on grain composition which must therefore be taken into account when comparing the compositions of grain samples.

## Methods

### Plant material

39 bread wheat cultivars were selected to represent diversity in UK adapted commercial wheats released and grown between 1790 and 2012 (Fig. 1). These were grown at Rothamsted Research in three replicate 1m^2^ plots for three successive seasons: 2013–2014, 2014–2015 and 2015–2016. Nitrogen was applied as ammonium nitrate at 210 kg/Ha (2013–2014) or 150 kg/Ha (2014–2015, 2015–2016) with other inputs being according to standard agronomic practice. Plots were staked where necessary and heads harvested and threshed by hand. Grain was conditioned to 16.5% water content and milled using a Chopin CD1 mill to give white flour.

### Genotyping

The Axiom Wheat HD Genotyping Array (Thermo Fisher Scientific, Inc., Waltham, MA) (comprising 816,571 SNP markers) was used to genotype the 39 samples using the Affymetrix GeneTitan (Thermo Fisher Scientific, Inc.) system according to the procedure described by Affymetrix (Life Technologies, 2017). Allele calling was performed using the Affymetrix proprietary software package Axiom Analysis Suite, following the Axiom Best Practices Genotyping Workflow. A distance matrix was generated from the genotype scores using R package SNPRelate^25^. The proportion of variance for the first six eigenvalues was as follows: 8.66, 6.58, 5.85, 5.10, 3.88, 3.68. The first two eigenvalues accounting for over 15% of the variance were plotted as a PCA plot.

### Arabinoxylan and β-glucan

Enzymatic fingerprinting of AX was as described previously^26^. White flour was digested using a mixture of endoxylanase and lichenase (β-glucanase) to release arabinoxylan oligosaccharides (AXOS) and gluco-oligosaccharides (GOS) comprising 3 and 4 residues (G3, G4), respectively. These were separated using a Carbopac PA-1 (Dionex) column with dimensions 2 mm × 250 mm and the flow rate of 0.25 mL/min based upon the original method of Ordaz-Ortiz *et al*.^27^. At least two technical replicates of each biological replicate were analysed. The areas under the AXOS peaks were combined to determine TOT-AX and under the G3 and G4 GOS peaks to give total β-glucan (expressed in arbitrary units).

### NMR spectroscopy

^1^H-NMR sample preparation was carried out according to the procedures described previously^28,29^. Flour samples (30 mg) were extracted 80:20 D_2_O:CD_3_OD containing 0.05% *d*_4_– trimethylsilylpropionate (TSP) (1 ml) as internal standard. ^1^H-NMR spectra were acquired under automation at 300 °K using an Avance Neo Spectrometer (Bruker Biospin, Coventry, UK) operating at 600.0528 MHz, equipped with a cryoplatform and a 5 mm triple resonance inverse (TCI) probe. Spectra were collected using a water suppression pulse sequence (zgpr) with a 90° pulse and a relaxation delay of 5 s. Each spectrum was acquired using 16 scans of 65,536 data points with a spectral width of 7143 Hz. Spectra were automatically Fourier-transformed using an exponential window with a line broadening value of 0.5 Hz. Phasing and baseline correction were carried out within the instrument software. ^1^H chemical shifts were referenced to d_4_-TSP at δ0.00.

^1^H-NMR spectra were automatically reduced, using Amix (Analysis of MIXtures software, BrukerBiospin), to ASCII files containing integrated regions or ‘buckets’ of equal width (0.01 ppm). Spectral intensities were scaled to the d_4_-TSP region (δ0.05 to −0.05). The ASCII file was imported into Microsoft Excel for the addition of sampling/treatment details. Signal intensities for characteristic spectral regions for 29 major metabolites were extracted via comparison to library spectra of known standards run in the same solvent system using equivalent NMR data acquisition and processing parameters.

### Statistical methods

Analysis of variance (ANOVA) was used to assess the effect of variety differences over the 3 experiments. Each field trial was an independent randomized complete block design so the ANOVA structure includes both a Year and Block within Year random effect. Two treatment structures were considered, i) Genotype/Time looking to assess variety differences and Variety.Time interactions and ii) (Group/Genotype) /Time where Group classifies varieties according to the year of introduction as defined in Fig. 1. Variables were transformed, as detailed in Table 2 and Supplementary Table S3, to ensure homogeneity of variance.

Multivariate analyses were used to assess variation across all variables. Fibre components were considered as a composition (relative percentage out of 100) and as such were first transformed according to the centred log ratio transformation^30^. To ensure subsequent multivariate analyses focussed on variation between cultivars, variables were adjusted by the Year.Block BLUPs before input into the PCA. PCA was done on the correlation matrix.

All statistical analyses were done using Genstat 20^th^ edition.

## Supplementary information

Supplementary data.

## Data Availability

All raw data used in the reported study has been made available in the Supplementary Files submitted.
