# Supplementary material for: Historical changes in the contents and compositions of fibre components and polar metabolites in white wheat flour
Source: Sci Rep. 2020 Apr 3;10:5920. doi: 10.1038/s41598-020-62777-3 (PMC7125105; doi:10.1038/s41598-020-62777-3)
Supplement: Supplementary file 1 — Supplementary data. [file 41598_2020_62777_MOESM1_ESM.zip › 2403/1 Supplementary Figure legends 100320.pdf]

Historical changes in the contents and compositions of fibre components and polar metabolites in white wheat flour.

Alison Lovegrove, Till K Pellny, Kirsty L Hassall, Amy Plummer, Abigail Wood, Alice Bellisai, Alexandra Przewieslik-Allen, Amanda J. Burridge, Jane L. Ward and Peter R Shewry.

Supplementary Figure Legends.

Figure S1. Helium plot of pedigrees for the 39 cultivars used in this study.

Figure S2. Box and whisker plots of the weight, hardness index and diameter of grain of the 39 cultivars grown in three years.

Cultivars are combined into three groups representing release dates (age groups) (see Table 1) and data presented are the means for each group grown in each year. Boxes delineate the upper and lower quartile. Whiskers represent upper and lower values and means are represented by a solid line within boxes. Error bar gives the least significance difference (LSD) between groups. Group average is indicated by a black asterisk (✷).
