# Supplementary material for: Historical changes in the contents and compositions of fibre components and polar metabolites in white wheat flour
Source: Sci Rep. 2020 Apr 3;10:5920. doi: 10.1038/s41598-020-62777-3 (PMC7125105; doi:10.1038/s41598-020-62777-3)
Supplement: Supplementary file 1 — Supplementary data. [file 41598_2020_62777_MOESM1_ESM.zip › 2403/13 Supplementary Table S4 Title page.pdf]

Historical changes in the contents and compositions of fibre components and polar metabolites in white wheat flour.

Alison Lovegrove, Till K Pellny, Kirsty L Hassall, Amy Plummer, Abigail Wood, Alice Bellisai, Alexandra Przewieslik-Allen, Amanda J. Burridge, Jane L. Ward and Peter R Shewry.

Supplementary Table S4. Cultivar means for each study year. Total and individual dietary fibre and polar metabolite components.
