# Supplementary material for: Historical changes in the contents and compositions of fibre components and polar metabolites in white wheat flour
Source: Sci Rep. 2020 Apr 3;10:5920. doi: 10.1038/s41598-020-62777-3 (PMC7125105; doi:10.1038/s41598-020-62777-3)
Supplement: Supplementary file 1 — Supplementary data. [file 41598_2020_62777_MOESM1_ESM.zip › 2403/6 Supplementary Table legends 100320.pdf]

Supplementary Table Legends

Table S1. Cultivar means for each study year: Single Kernel Characterisation System (SKCS) measurements; grain kernel weight, diameter and hardness index.

Least Squares Difference (LSD) shown is the LSD between cultivars within the same year.

Table S2. Raw data collected from the 39 cultivars grown in three years in randomised field trials in the UK used in PCA analysis.

Table S3. ANOVA of the treatment effects for individual variables.

All treatment effects were tested on 221 residual degrees of freedom. Table shows both the F statistic (representing the size of effect) and the p-value (the statistical significance of the effect). Individual fibre and polar metabolites are listed. Fibre components are total AX (relative peak area) total beta-glucan (relative peak area) and as % peak areas of total. Polar metabolites as mg/g.

Table S4. Cultivar means for each study year: Total and individual dietary fibre and polar metabolite components.

Least Squares Difference (LSD) shown is the LSD between cultivars within the same year. Means are shown on the scale of analysis, i.e. on the scale of transformation as indicated in the column labels.
