# Supplementary figures and images for: Historical changes in the contents and compositions of fibre components and polar metabolites in white wheat flour
Source: Sci Rep. 2020 Apr 3;10:5920. doi: 10.1038/s41598-020-62777-3 (PMC7125105; doi:10.1038/s41598-020-62777-3)

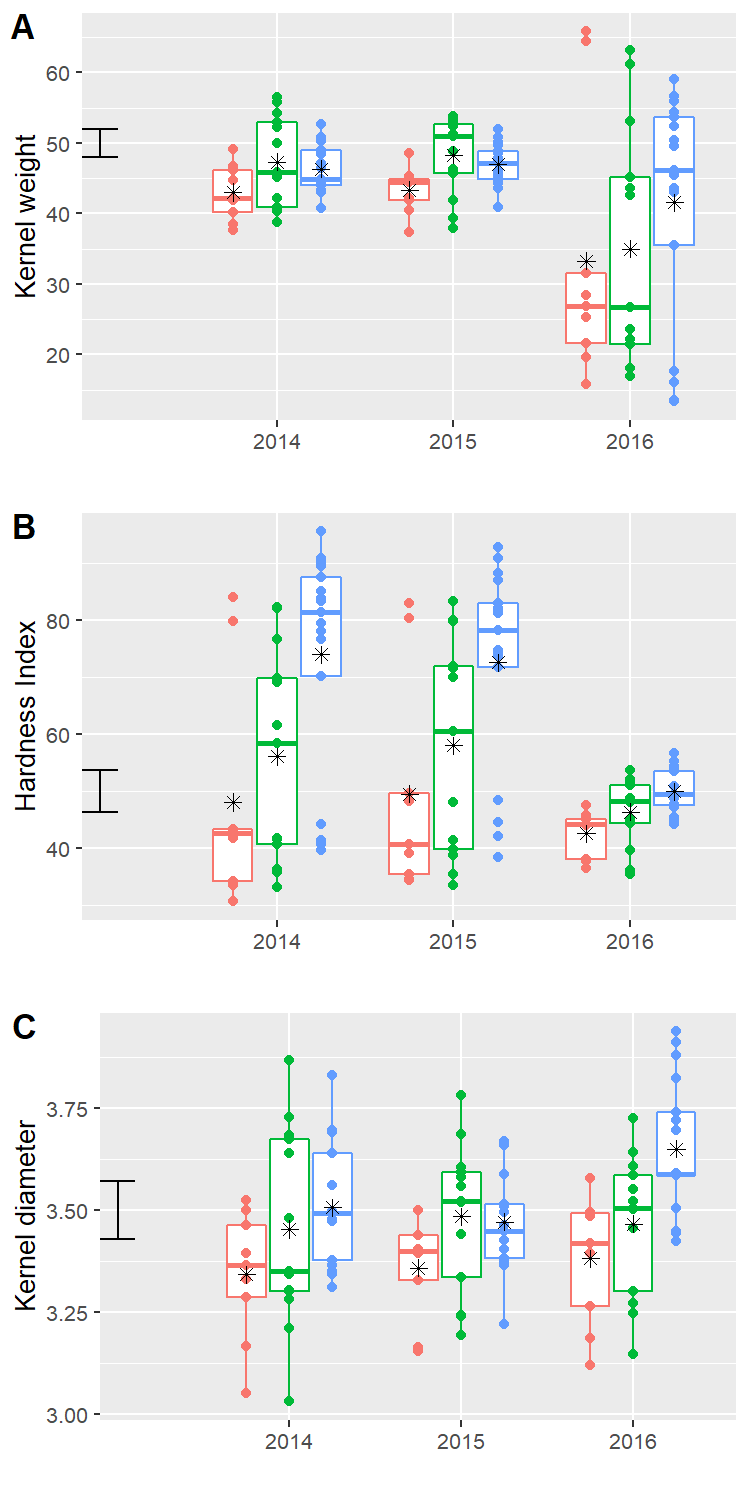

Supplement: Supplementary file 1 — Supplementary data. [file 41598_2020_62777_MOESM1_ESM.zip › 2403/5 Supplementary Figure S2 100320.tif]
